# Supplementary material for: Overexpression of AtBBD1, Arabidopsis Bifunctional Nuclease, Confers Drought Tolerance by Enhancing the Expression of Regulatory Genes in ABA-Mediated Drought Stress Signaling
Source: Int J Mol Sci. 2021 Mar 13;22(6):2936. doi: 10.3390/ijms22062936 (PMC8001636; doi:10.3390/ijms22062936)
Supplement: Supplementary file 1 [file ijms-22-02936-s001.pdf]

Supplementary Materials

Overexpression of *AtBBD1*, Arabidopsis bifunctional nuclease, confers drought tolerance by enhancing the expression of regulatory genes in ABA-mediated drought stress signaling

A K M Mahmudul Huque [1], Wonmi So [1], Minsoo Noh [1], Min Kyoung You [2,*] and Jeong Sheop Shin [1,*]

[1] Division of Life Sciences, Korea University, Seoul 02841, Korea; mahmudornob@korea.ac.kr (A.K.M.M.H.); thso1124@gmail.com (W.S.); redmoon89@korea.ac.kr (M.N.)
[2] Graduate School of Biotechnology, Kyung Hee University, Yongin 446-701, Korea
* Correspondence: zinggy93@gmail.com (M.K.Y); jsshin@korea.ac.kr (J.S.S)

Table S1. List of primers used in this study

Experiments	Name	Sequence 5′→3′
Cloning of overexpression construct	BBD1_OX	F: TGC**TCTAGA** ATGAGGTCGCTTCAAGCACCG
		R: CGC**GGATCC**TCATGTGTATTTCCTTAATTTGCGTTTTGCC
Cloning of sGFP construct	BBD1-sGFP	F: TGC**TCTAGA** ATGAGATCGGTTCAAGCACCAGTTG
		R: CGC**GGATCC**TCATGTGTATTTCCTCAAGTTACGTTTGGCC
	BBD2-sGFP	F: TGC**TCTAGA** ATGAGGTCGCTTCAAGCACCG
		R: CGC**GGATCC**TCATGTGTATTTCCTTAATTTGCGTTTTGCC
Cloning of Promoter:GUS construct	Pro_{BBD1}:GUS	F: CCC**AAGCTT**ACAAGTTCCAGACACGAGCACCG
		R: CGC**GGATCC**CTTCCTTCCTTCAATCAAACAGAGTATTTTAGTGT
	Pro_{BBD2}:GUS	F: CCC**AAGCTT**CACATTTAAATAGCTATATAATTCTCTATATAATTCTTA
		R: CGC**GGATCC**CTTCACTTTTTGATGCAGAACCCAGAAA
RT-PCR and qRT-PCR	BBD1	F: AAGACCGACCAAATGGTCAGGC
		R: CAAGTTACGTTTGGCCCGAAACTGAC
	BBD2	F: GGTATGATGAAGCCGCTGAGTG
		R: CTTCTGGCAAATGGCAATGGCTTC
	PP2AA3	F: CCAAACATCAAATTTAACGTGGCCAAAATG
		R: GTTCTCCACAACCGCTTGGT
	eIF4a1	F: GCTCTCCCGTGGTTTCAAGGACCAGATC
		R: GTCTGTGAGCCAATCAACCTTACGCCTG
	P5CS1	F: AGAGTCAATGGTGGCTCGCTTAGT
		R: ACACGGCCGATTGGATCTTCCATA
	PRODH	F: ATTTGGCAGTACAAAATGCT
		R: AGGACAATGCATCTGACATA
	ABA1	F: CGACGATTGTTGCGTTTCGG

		R: GCATGCATCTTCGAAACCTGAG
	NCED3	F: ACATGGAAATCGGAGTTACAGATAG
		R: AGAAACAACAAACAAGAAACAGAGC
	RD29B	F: TACCTTCCGACCAGATAGCGG
		R: CGAAAACCCCATAGTCCCAACG
	RAB18	F: CCAGCTCTAGCTCGGAGGA
		R: GACTGATCATGATGACCTGGCAAC
	RD20	F: TGGTTTCCTATCTAAAGAAGCTGTG
		R: ATACAAATCCCCAAACTGAATAACA
	RD22	F: GCTGTTTCCACTGAGGTGGC
		R: ATGAGTCTCCGGGAGGAAGTG
	MYB44	F: AATGGGGAAGTCTTTTCCCGG
		R: CCGTTGCATCTCCGTCATGTAAC
	ERD10	F: GCTCTTCTTCCTCTTCGAGTGATG
		R: CCACTGTTTTCACATGATCTCCTTC
	PP2CA	F: GCGGCGTTGCTCTTAACAAAG
		R: GACGACGCTTGATTATTCCTCCTC
	CYP707A3	F: CATGCCTTTTGGTAGTGGGA
		R: CGATTGACCATCTGTACTTAGTGG
	NAC019	F: AACTGTGGCTACCTGAAGAC
		R: CCGAGTTATTAAACCCGTGA
	ZAT10	F: AGGCTCTTACATCACCAAGATTAG
		R: TACACTTGTAGCTCAACTTCTCCA
	LEA4-5	F: GTGACCGACCCGATTGGAAG
		R: CCCCGCCGGTTCCGTACCCG
	LTP3	F: GTGGGTTGGTGCCACCTTCA
		R: TCACTTGATGTTGTTGCAGT
	MYC2	F: ACCACGTCGAAGCAGAGAGACAAA
		R: TGTAAGCGATTGCGTCACCGAGTA

* The bold underlined letters indicate restriction enzyme recognition sites. F, forward primer; R, reverse primer

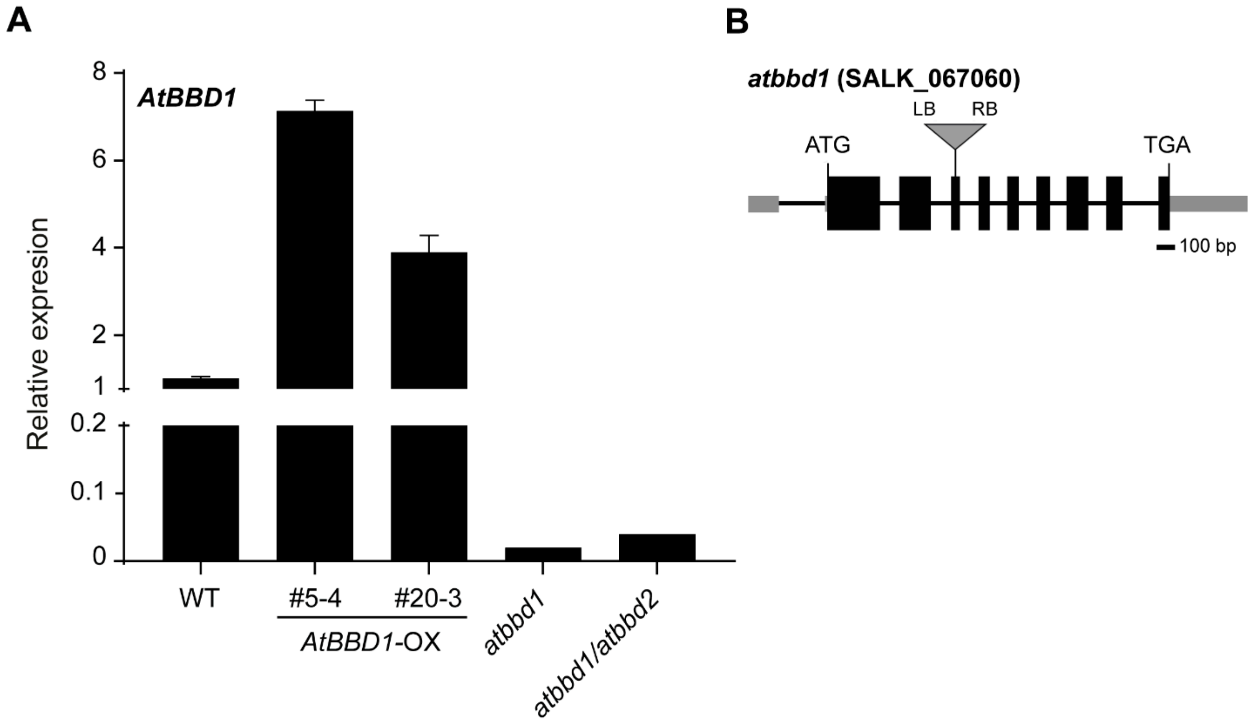

Figure S1. Characterization of *AtBBD1* overexpression (OX), *atbbd1* and *atbbd1/atbbd2* mutant lines. **(A)** qRT-PCR analysis of *AtBBD1* expression in the WT, two independent *AtBBD1*-OXs, *atbbd1,* and *atbbd1/atbbd2* plants. Two-week-old Arabidopsis (Col-0) plants grown on 1/2 MS agar media were used for qRT-PCR assay. The relative gene expression was calculated and normalized to the reference gene *eIF4a1*. The normalized mRNA levels in wild-type plants were set to 1. Data represent the mean ± standard error values of three independent experiments. **(B)** T-DNA map of *atbbd1* (SALK_067060).

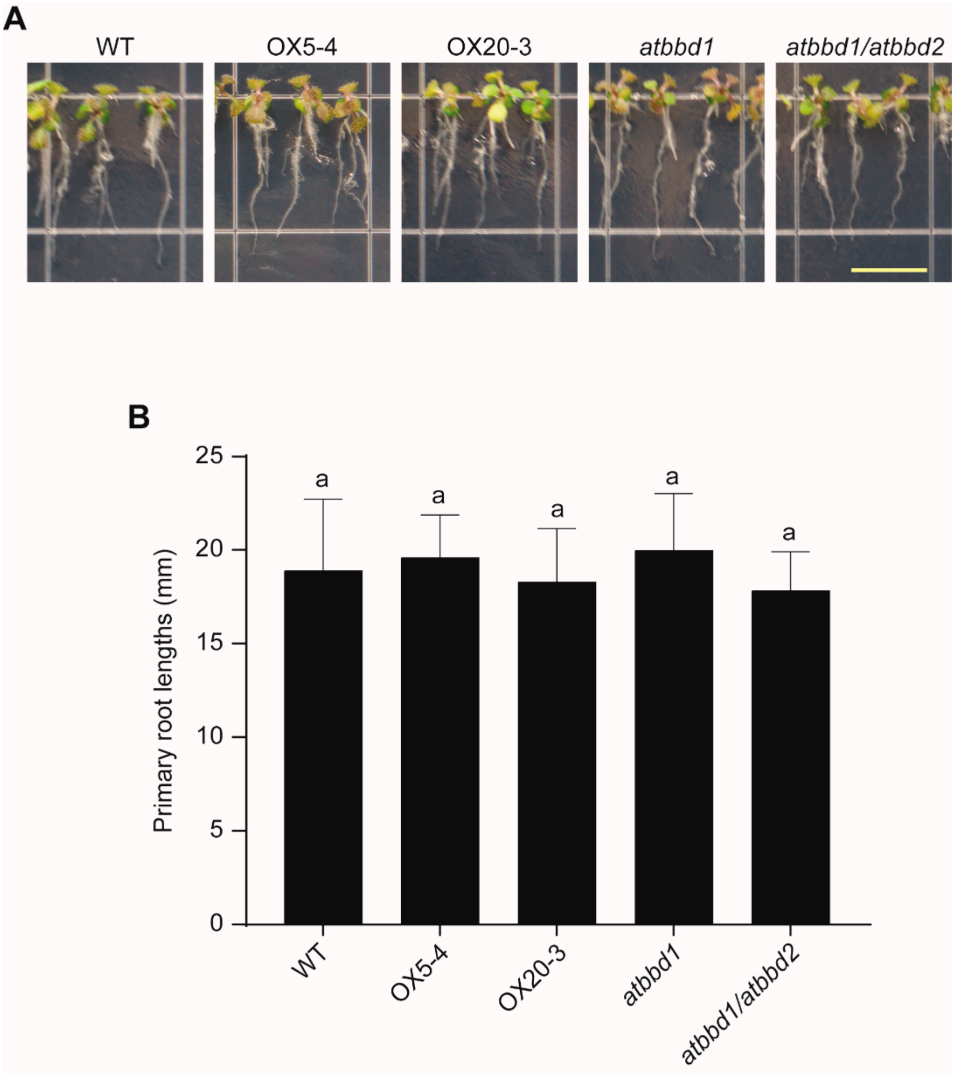

Figure S2. Root phenotype of the WT, OX5-4, OX20-3, *atbbd1* and *atbbd1/atbbd2* plants in response to MeJA. **(A)** Primary root growth of the WT, OX5-4, OX20-3, *atbbd1* and *atbbd1/atbbd2* plants on 1/2 MS agar plates containing 20 µM MeJA. Representative photographs were taken 7 days after plating. Scale bar = 10 mm. **(B)** Measurement of primary root lengths of five lines in (A). Bars represent the mean ± SD of 12 seedlings of each genotype. The same letter "a" indicates that root lengths of five lines are not significantly different (ANOVA; $P<0.05$).

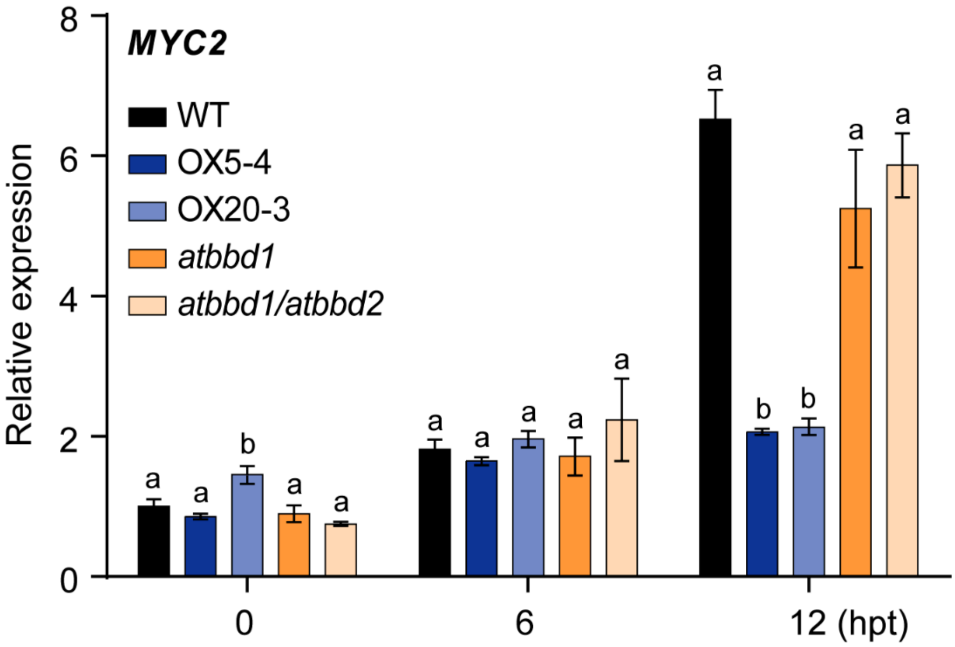

Figure S3. Expression pattern of *MYC2* in the WT, OX5-4, OX20-3, *atbbd1* and *atbbd1/atbbd2* lines under the drought stress conditions by qRT-PCR. Three-week-old soil-grown plants were watered with 20% PEG 6000 and qRT-PCR of *MYC2* expression was analyzed at designated time points. The relative gene expression was calculated and normalized to the reference gene *eIF4a1*. Data represent the mean ± standard error of three independent experiments. One-way ANOVA with post-hoc Tukey test was used for the statistical comparison of all genotypes. Different letters indicate significant differences among genotypes at each time point ($P < 0.05$).

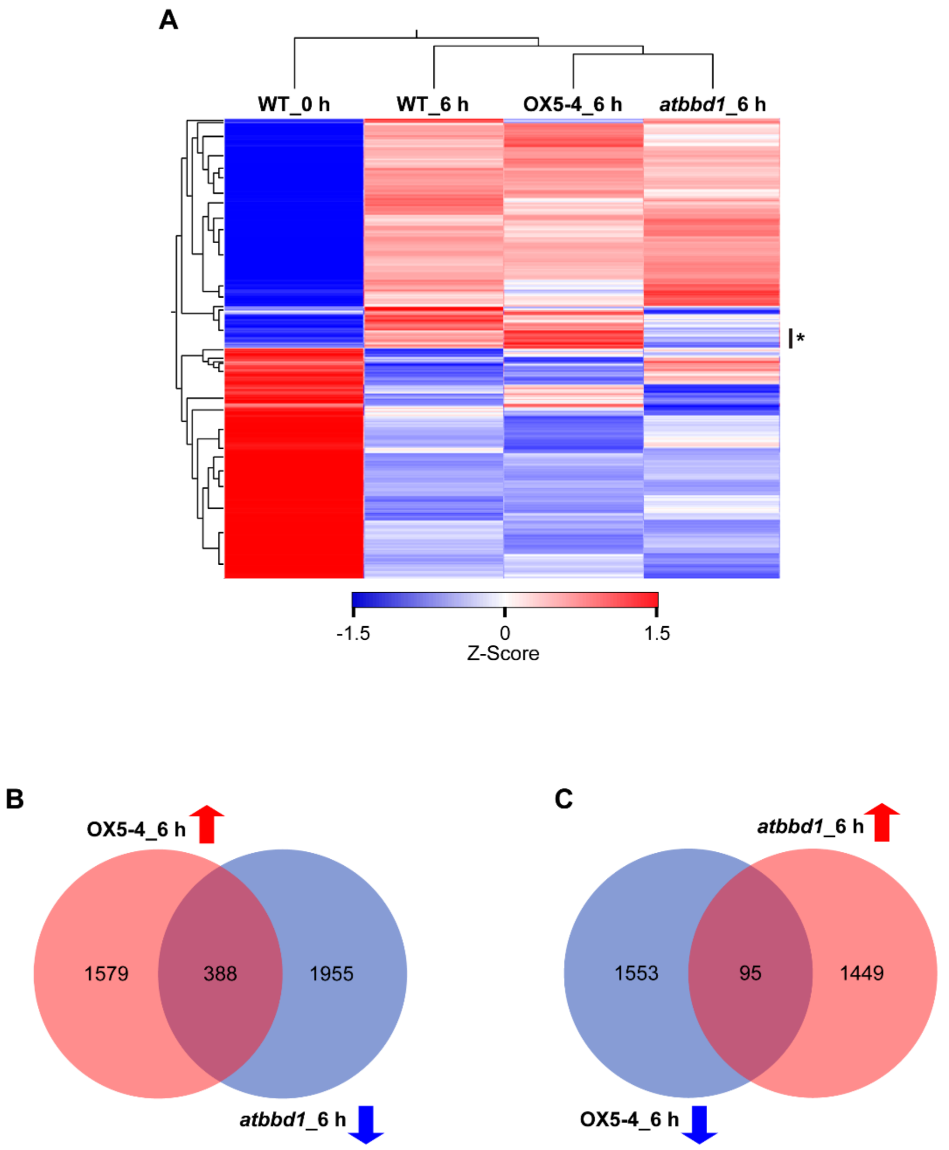

Figure S4. Transcriptome analysis of the WT, OX5-4, and *atbbd1* lines under the drought stress conditions. Three-week-old soil-grown plants were watered with 20% PEG 6000 and then RNA-seq was analyzed with samples collected after 6 h of treatment. **(A)** Heatmap of RNA-seq expression z-scores computed for all genes that were differentially expressed ($\log_2$(foldchange) > 1) among all pairwise comparisons of the WT, OX5-4 and *atbbd1* plants under drought stress conditions with the WT grown under well-watered conditions. Each column in the heatmap is an individual sample. **(B)** Venn diagram of common and unique DEGs that were up-regulated in OX5-4 and down-regulated in *atbbd1* compared to the WT under the drought stress conditions. **(C)** Venn diagram of common and unique DEGs that were down-regulated in OX5-4 and up-regulated in *atbbd1* compared to the WT under the drought stress conditions.

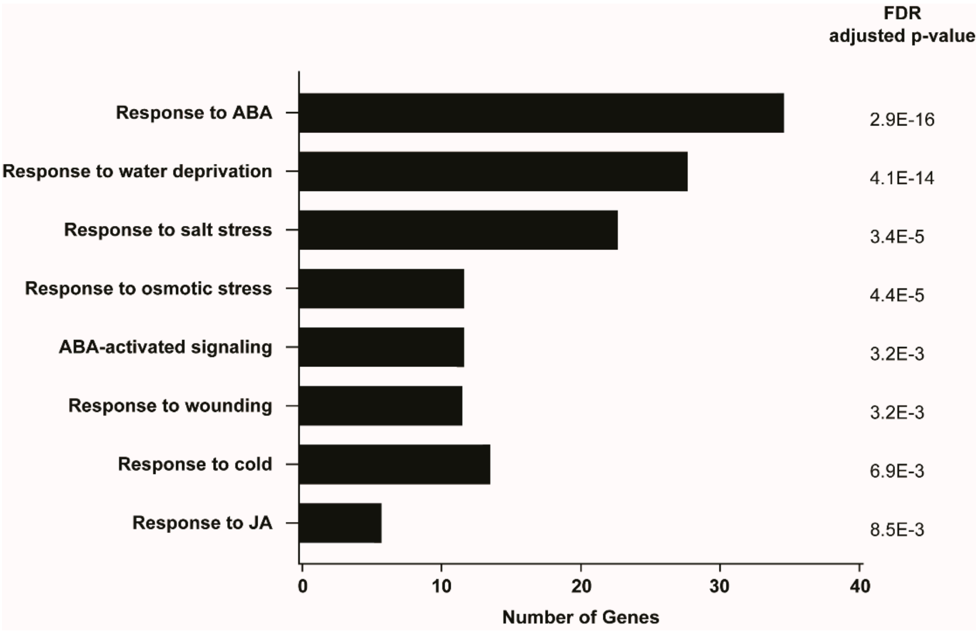

Figure S5. Gene ontology analysis of 388 differentially expressed genes that were up-regulated in OX5-4 and down-regulated in *atbbd1* compared to the WT under the drought stress conditions. The biological processes showing the FDR (false discovery rate) adjusted p-value ≥ 0.05, calculated by the Benjamini's method, were used in this analysis.

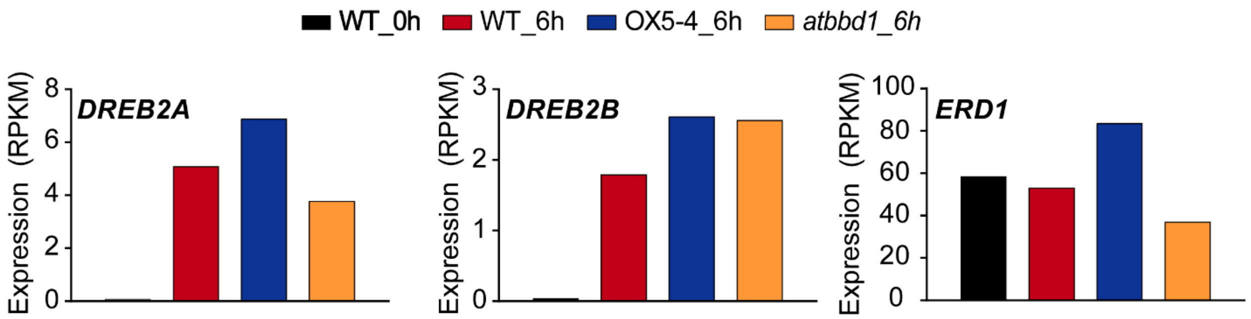

Figure S6. Expression of genes related to ABA-independent signaling in response to drought stress. RPKM levels of *DREB2A, DREB2B* and *ERD1* were determined by RNA-seq in the WT, OX5-4 and *atbbd1* plants under drought stress condition. Samples were collected from three-week-old soil-grown plants after 6 h of stress from watering with 20% PEG 6000.
